# Impaired Representation of Geometric Relationships in Humans with Damage to the Hippocampal Formation

**DOI:** 10.1371/journal.pone.0019507

**Published:** 2011-05-18

**Authors:** Carsten Finke, Florian Ostendorf, Mischa Braun, Christoph J. Ploner

**Affiliations:** Klinik für Neurologie, Charité – Universitätsmedizin Berlin, Berlin, Germany; University of Alberta, Canada

## Abstract

The pivotal role of the hippocampus for spatial memory is well-established. However, while neurophysiological and imaging studies suggest a specialization of the hippocampus for viewpoint-independent or allocentric memory, results from human lesion studies have been less conclusive. It is currently unclear whether disproportionate impairment in allocentric memory tasks reflects impairment of cognitive functions that are not sufficiently supported by regions outside the medial temporal lobe or whether the deficits observed in some studies are due to experimental factors. Here, we have investigated whether hippocampal contributions to spatial memory depend on the spatial references that are available in a certain behavioral context. Patients with medial temporal lobe lesions affecting systematically the right hippocampal formation performed a series of three oculomotor tasks that required memory of a spatial cue either in retinal coordinates or relative to a single environmental reference across a delay of 5000 ms. Stimulus displays varied the availability of spatial references and contained no complex visuo-spatial associations. Patients showed a selective impairment in a condition that critically depended on memory of the geometric relationship between spatial cue and environmental reference. We infer that regions of the medial temporal lobe, most likely the hippocampal formation, contribute to behavior in conditions that exceed the potential of viewpoint-dependent or egocentric representations. Apparently, this already applies to short-term memory of simple geometric relationships and does not necessarily depend on task difficulty or integration of landmarks into more complex representations. Deficient memory of basic geometric relationships may represent a core deficit that contributes to impaired performance in allocentric spatial memory tasks.

## Introduction

Spatial behavior critically depends on representation of a subject's current action targets and of the environmental layout that is interacted with. Multiple lines of evidence have suggested that the hippocampus is pivotal for the formation of corresponding representations [1]–[4]. A central hypothesis of many animal and human studies holds that the hippocampus is particularly important for memory of allocentric, i.e. viewpoint-independent spatial information [1], [5]. Possible neural correlates for this type of representation have recently been identified in the human hippocampus by means of single-neuron recordings and functional imaging in subjects performing virtual navigation tasks [6]–[8].

Patient studies have shown that humans with lesions affecting the hippocampus are impaired across a wide range of visuo-spatial memory tasks [9]–[20]. This deficit appears particularly robust in conditions in which the spatial relationship between subject and visuo-spatial layout is manipulated during the memory delay [9]–[15]. Although this procedure is intended to force subjects to maintain viewpoint-independent spatial relationships, it has proven difficult to relate deficits in patient studies to the concept of allocentric memory. First, it has been argued that even in studies that used control conditions encouraging egocentric, i.e. viewpoint-dependent strategies, differences in task difficulty may explain a disproportionate impairment in allocentric conditions [21]. Second, simultaneous encoding of egocentric and allocentric information may confound the interpretation of behavioral deficits in tasks that employ changes in the spatial relationship between subject and memoranda [22], [23]. Third, learning of complex spatial layouts is likely to require integration of diverse information such as geometry, landmarks and their associations. Animal and human studies suggest the possibility that these types of information are represented in distinct systems [24]–[26].

Here, we have investigated whether hippocampal contributions to spatial memory depend on the spatial references that are available in a certain behavioral context. Patients with medial temporal lobe lesions affecting systematically the right hippocampal formation performed a series of oculomotor tasks that required memory of a spatial cue either in retinal coordinates or relative to a simple environmental reference. Availability of these references was strictly controlled. In addition, stimulus displays were free of the complex visuo-spatial associations that are frequently present in spatial memory tasks. We aimed to identify deficits that may underlie deficient performance in allocentric spatial memory tasks.

## Materials and Methods

### Subjects

**Figure 1:**
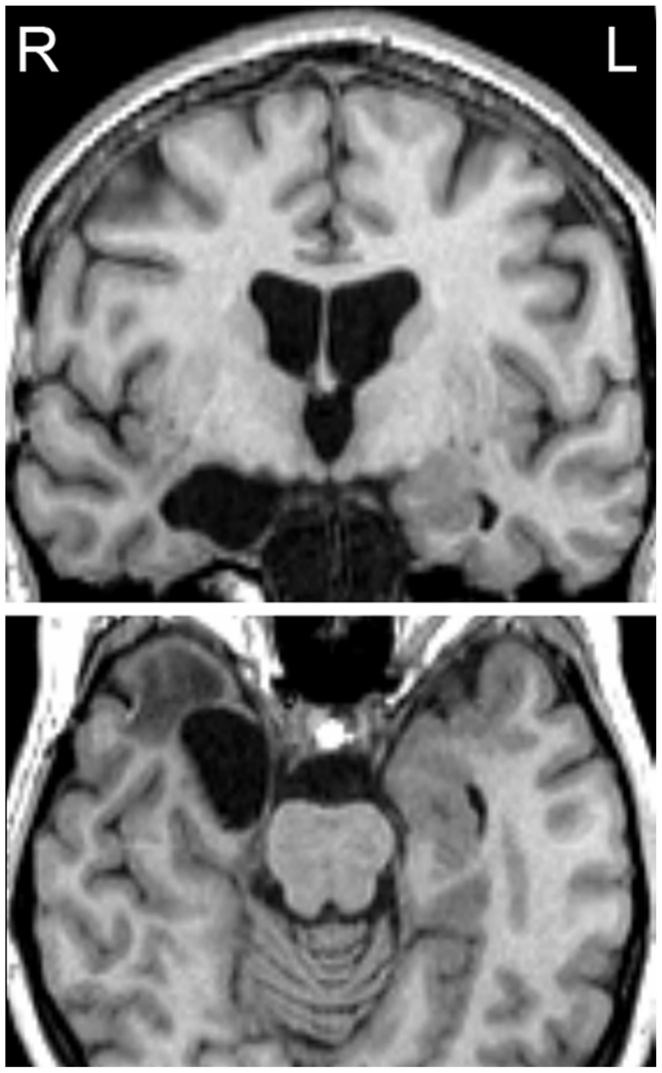
Example lesion, patient H.N. Top: coronal MRI section perpendicular to the line connecting the anterior and posterior commissures (AC-PC line), at the level of amygdala, hippocampal head, rostral entorhinal cortex, rostral perirhinal cortex and infero-temporal cortex. Bottom: Axial MRI section parallel below the AC-PC line, at the level of amygdala, rostral hippocampus, entorhinal cortex, perirhinal cortex and infero-temporal cortex. Note damage to rostral hippocampus and adjacent MTL structures on the right.

Five patients (three females, two males; mean age 28 years, range 21–42 years) were recruited from the Department of Neurosurgery at the Charité-Universitätsmedizin Berlin, Germany. All had undergone resection of right MTL structures for the treatment of benign brain tumors causing epilepsy (figure 1). All patients had already participated in previous experiments in our laboratory (patients H.N., A.M., S.W., S.D. and D.B., (see Braun et al. [27], [28] and Finke et al. [20] for MRIs and individual patient characteristics). Seizures had ceased post-operatively in all patients and they were fully integrated in their professional and social lives. Patients were free of additional neurological or psychiatric disorders and had normal or corrected-to-normal vision. All patients received anticonvulsant medication in regular dosages. The control group consisted of 10 healthy subjects (six females, four males; mean age 29 years, range 25–37 years; no significant difference between patient and control group; p = 0.59) without any history of neurological or psychiatric disorders.

### Ethics statement

Informed written consent was obtained from each subject before participation in the study that was approved by the ethics committee of the Charité – Universitätsmedizin Berlin and conducted in conformity with the Declaration of Helsinki.

### Lesion evaluation

In patients, structural magnetic resonance imaging was performed in a Philips 1.5 T scanner with a three-dimensional gradient echo sequence to obtain isotropic volume elements of 1 mm^3^. Covering the temporal lobes, 80–100 coronal sections of 1 mm thickness each were reconstructed in perpendicular orientation to the line connecting anterior and posterior commissures. Individual extent of damage to different sub-regions of the MTL (amygdala, hippocampus, entorhinal cortex, perirhinal cortex, parahippocampal cortex and infero-temporal cortex) was then determined by identifying anatomical landmarks according to Braun et al. [27]. All patients had damage to the right amygdala, anterior hippocampus, anterior entorhinal cortex and portions of perirhinal cortex. One patient (D.B.) had slight additional damage to the anterior parahippocampal cortex and in another patient (S.D.), parahippocampal and inferotemporal cortices were affected by the neurosurgical operation. In the three remaining patients, parahippocampal and inferotemporal cortices were intact (see Braun et al. [27], [28] and Finke et al. [20] for individual lesion characteristics).

### Experimental Setup

Subjects were seated in front of a 22 in. CRT-monitor (refresh-rate 110 Hz) in a completely darkened room. The head was positioned on a chinrest to ensure a constant viewing distance of 50 cm to the screen (43.6×33.4° of visual angle). In front of the monitor, an 80×80 cm (32×32 in.) semi-translucent acrylic glass pane was mounted. The pane masked the frame of the monitor during experiments and covered a visual angle of 72×72°. Visual stimuli were thus presented on a homogenous gray plane without any further references. Stimuli were programmed and presented with ERTS software (Version 3.32; BeriSoft, Frankfurt, Germany). Luminance of stimuli as measured through the pane was 1.4 Cd/m^2^ for memory cues and 1.1 Cd/m^2^ for reference bars (see below). Monitor background luminance was <0.001 Cd/m^2^. Movements of the right eye were recorded by means of video-oculography at a sampling rate of 500 Hz (iView Hi-Speed, Sensomotoric Instruments, Teltow, Germany).

### Paradigms

Subjects were tested in three variants of a classic oculomotor short-term memory paradigm (“memory-guided saccade task”) termed EGO, ALLO-EGO and ALLO. In all three variants, subjects had to remember the location of a memory cue and, after an unfilled delay, make a saccade based on their memory of the cue location. Although the temporal structure and stimulus characteristics of the tasks were identical, tasks differed in availability and significance of visual references. Subjects were thus forced to remember cue positions either in retinal coordinates or relative to a single environmental reference. Our rationale was to control a subjects' use of spatial reference frames as strictly as possible with sole reliance on egocentric memory in the EGO condition, on allocentric memory in the ALLO condition and reliance on both in the ALLO-EGO condition.

**Figure 2:**
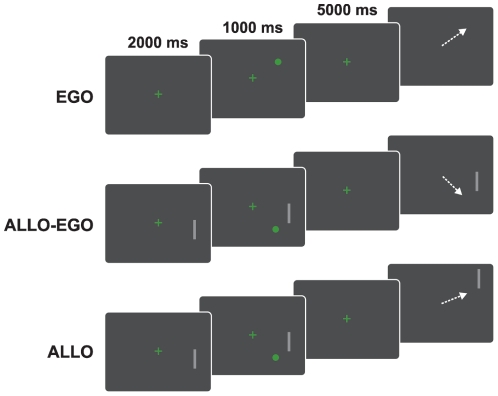
Schematic of the tasks. EGO task: Subjects were presented a memory cue while fixating on a central fixation cross. After an unfilled delay of 5000 ms, subjects performed an eye movement to the remembered cue position (‘memory-guided saccade’). ALLO-EGO: While fixating on a central fixation cross, subjects were presented a bar, followed by presentation of a memory cue together with the bar. After an unfilled delay of 5000 ms, the bar re-appeared and subjects performed an eye movement to the remembered cue position. ALLO: This task was identical to the ALLO-EGO task until the end of the delay. Then, the bar re-appeared at a new location. Subjects performed an eye movement to the relative position of the cue with respect to the bar (i.e. to the position of the memory cue if it had been displaced together with the bar).

In all tasks, memory cues (green filled circles, size: 0.5° of visual angle) were presented in one of 12 possible locations (three locations in each quadrant of the screen). Cue locations were arranged on imaginary circles around a central fixation cross (size: 0.5° of visual angle) at a radius of 8° (4 positions) and 12° (8 positions). 8°-cue positions were located 30° above and below the horizontal midline, 12°-cue positions were located 20° and 40° above and below the horizontal midline (figure 2). In the ALLO-EGO and in the ALLO tasks, a horizontal or vertical bar (light-grey, length: 6°) was presented together with the memory cue in one of eight possible locations (two locations in each quadrant of the screen). Bars were presented either at 9° (horizontal bar) or 13.5° (vertical bar) radial distance to the central fixation cross (figure 2).

#### EGO

The memory cue was presented for 1000 ms while subjects fixated on the central fixation cross. Subjects were instructed to maintain central fixation and remember the cue location for a subsequent delay of 5000 ms. The offset of the fixation cross at the end of the delay served as the signal to make a memory-guided saccade to the remembered cue position.

#### ALLO-EGO

A bar was presented for 2000 ms while subjects fixated on the central fixation cross. 1000 ms after bar onset, a memory cue was presented in the same quadrant for 1000 ms. During the subsequent delay of 5000 ms, subjects maintained central fixation and remembered the cue location. After the delay, the bar reappeared and subjects made a saccade to the remembered cue position.

#### ALLO

This task was identical to the ALLO-EGO-task except for the fact that after the delay, the bar reappeared at a different location. Subjects were instructed not to make a saccade to the remembered cue position but to the relative position of the cue with respect to the displaced bar (i.e. the position the memory cue would occupy if it had been displaced together with the bar). The locations of the displaced bars were calculated so that “new” cue locations matched one of the 12 memory cue locations. Stimulus configurations that would result in a location of the displaced bar between the central fixation cross and the “new” cue location were avoided (e.g. a shift of the horizontal bar from the 20°-position in the upper right quadrant to the 40°-position in the lower right quadrant; figure 1).

### Procedure

The experiment was run in a blocked design with separate administration of the three tasks. Task order was counterbalanced across subjects. Each task consisted of 3 blocks with 16 trials arranged in pseudo-random order. In the ALLO-EGO and the ALLO tasks, each memory cue position was used twice with horizontal and twice with the vertical bar. Subjects were given time to rest between blocks. Breaks of about 30 min. duration were scheduled between tasks to avoid fatigue. Prior to each task, subjects performed 10 training trials. Two days of testing were required in each participant for completion of experiments.

### Data Analysis

Eye movement data were low-pass filtered, visualized and analyzed by using MATLAB (The Mathworks, Natick, MA), the ILAB toolbox [29] and self-written routines. Saccade onset was determined by using a fixed velocity criterion (threshold: 30 deg/s). Trials with eye movements exceeding 1° during stimulus presentation or delay period were excluded from further analysis (5.8% of trials in patients, 5.1% of trials in controls, p = 0.55). Saccade accuracy was described as systematic and variable amplitude error according to White et al. [30]. Systematic error of saccades was obtained by computing the distance between the target location and the saccadic end point by using the formula:
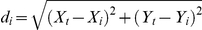

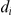
 = distance of a saccadic end point from target location

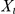
 = horizontal target position

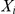
 = horizontal end position for saccade *i*

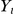
 = vertical target position

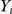
 = vertical end position for saccade *i*

For calculation of the variable error of saccades the average horizontal and vertical saccade landing position for the three targets of each quadrant were computed. Then, the absolute distance of each individual end point from the computed average end point was obtained by using the formula:
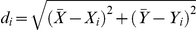

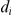
 = distance of a saccadic end point from the average end point

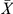
 = average horizontal end position

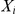
 = horizontal end position for saccade *i*

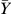
 = average vertical end position

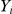
 = vertical end position for saccade *i*

Medians were used to describe individual systematic and variable errors in each task. As the number of subjects permitted no meaningful conclusions on the normality of the data distribution, non-parametric statistical analyses with Wilcoxon-tests, Mann-Whitney-tests and Friedman-ANOVAs were applied throughout [31].

## Results

**Figure 3:**
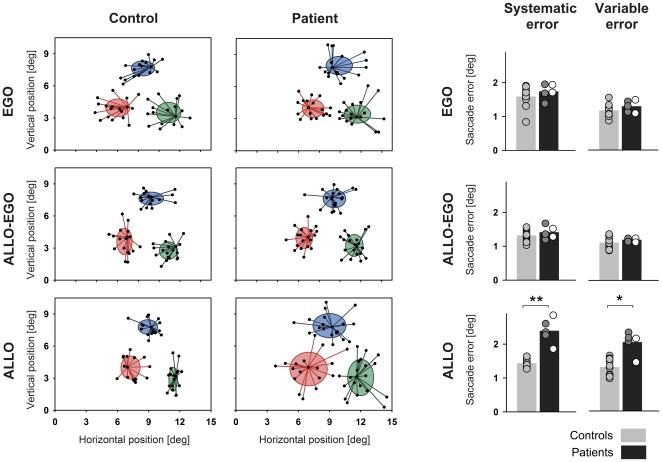
Example and group results. LEFT: Example results of a control subject and a patient (H.N.) in the EGO task (top row), ALLO-EGO task (middle row) and ALLO task (bottom row). Data are shown separately for the three memory cue positions, collapsed over all quadrants of the visual field (red, 8°/30°-position; green 12°/20°-position; blue, 12°/40° position). Filled black circles denote saccade end points. Ellipses are centered on a subjects' average saccade end point for a given memory cue position. Ellipse extent represents the horizontal and vertical standard deviation of saccade end points. Note decreased accuracy of the patients' saccade end points in the ALLO task. RIGHT: Group results. Mean systematic and variable saccade amplitude error in controls (light grey bars) and patients (dark grey bars) in the EGO task (top row), ALLO-EGO task (middle row) and ALLO task (bottom row). Dots represent individual performance. In patients, white dots represent performance of patients D.B. and S.D. (i.e. patients with involvement of parahippocampal cortex) and grey dots performance of patients H.N., A.M. and S.W. (i.e. patients without involvement of parahippocampal cortex). Asterisks indicate significant differences between groups (* p<0.01, ** p<0.001).

Example results from individual subjects and group results are shown in figure 3. In controls, no significant differences between tasks were found for systematic saccade errors (*d*f = 2, χ^2^ = 3.80, p = 0.15) and variable saccade errors (*d*f = 2, χ^2^ = 0.60, p = 0.74). However, in patients, significant differences between tasks were observed (systematic error, *d*f = 2, χ^2^ = 8.40, p<0.01; variable error, *d*f = 2, χ^2^ = 7.60, p<0.02). Compared to controls, patients showed significantly increased saccade errors selectively in the ALLO task (systematic error, p<0.001; variable error, p<0.01). This increase remained significant even when the two patients with additional parahippocampal damage (patients D.B. and S.D.) were removed from analysis (systematic error, p<0.01; variable error, p<0.01). Inspection of figure 3 further shows that their performance was similar to the performance of patients with more restricted medio-temporal damage (patients H.N., A.M. and S.W.). The group differences between patients and controls were found for stimuli from both hemifields (right: systematic error, p = 0.001; variable error, p = 0.001; left: systematic error, p = 0.03; variable error, p = 0.01). By contrast, no significant group differences were observed in the ALLO-EGO task (systematic error, p = 0.51; variable error, p = 0.25) and EGO task (systematic error, p = 0.25; variable error, p = 0.21).

Normal performance in the EGO and ALLO-EGO tasks shows that oculomotor performance per se was not compromised in our patients and that the mere presence of a potentially distracting visual stimulus, i.e. the reference bar in the ALLO-EGO task, did not significantly influence our patients' saccade accuracy. Likewise, impairment of a decision or preparation process prior to saccade execution is unlikely to account for deficient performance of patients in the ALLO task. In this case, we would have expected differences in saccadic reaction times between groups in the ALLO condition. However, saccade latencies did not differ significantly between patients and controls in any of the tasks (EGO, patients, 340 ms, controls, 347 ms, p = 0.31; ALLO-EGO, patients, 371 ms, controls, 426 ms, p = 0.44; ALLO, patients, 509 ms, controls, 499 ms, p = 0.77).

Since the ALLO task necessarily required representation of a viewpoint-independent spatial relationship, our patients' selective performance decrease may theoretically have resulted from an averaging process between an egocentric target representation (corresponding to the initial position of the memory cue) and an allocentric target representation (corresponding to the position the memory cue would have occupied if it had been displaced together with the bar). Mean displacement of the memory cue position in the ALLO task was 13.16±1.0°. When computed relative to the initial (egocentric) cue position, the systematic targeting error of patients' saccades in the ALLO task was of similar magnitude and amounted to a mean of 14.0±1.0°. A substantially smaller systematic targeting error (mean: 2.39±0.17°, see figure 3) thus indicates that saccades landed much closer to the new (allocentric) target position than to a position that would have resulted from averaging of egocentric and allocentric representations. Together with the significant increase in variable errors, these results therefore suggest that our patients' deficits resulted from a degraded representation of the geometric relationship of the memory cue relative to the reference bar.

## Discussion

We investigated the role of spatial references for hippocampal contributions to spatial memory. Human patients with medial temporal lobe lesions affecting systematically the right hippocampal formation showed a selective impairment in a new oculomotor task that required memory of a spatial cue relative to a single environmental reference across a delay of 5000 ms. Performance in conditions that allowed for memory of a spatial cue relative to subjects' body coordinates was not affected. These findings have two implications. First, they show that it is possible to assess hippocampal integrity with comparatively simple oculomotor paradigms. Second, they demonstrate the existence of a spatial deficit that has not yet been described in previous human lesion studies.

### Egocentric and allocentric memory in patients with hippocampal damage

Following the formulation of the hypothesis that the hippocampus represents space relative to environmental references [5], research as diverse as single-neuron recordings, lesion studies, neurodevelopmental investigations and imaging studies in animals and humans has converged on the idea that spatial memory is not a single entity, but rather consists of multiple distinct and interacting representations (see [1], [22]–[24] for reviews). It is however a matter of ongoing debate whether some representations particularly depend on the hippocampus or whether disproportionate impairment of one class of representations with hippocampal damage merely reflects factors related to the experimental design of the studies. For example, several researchers have observed selective deficits of hippocampal patients in tasks that were designed to assess viewpoint-independent spatial memory (e.g. [12], [14], [15], [32]). By contrast, a recent study concluded that shifting a subjects' viewpoint during the memory delay of an object-location task decreased performance regardless of hippocampal integrity, whereas increasing memory load led to a clear impairment in adult patients with acquired hippocampal damage [21]. It was concluded that the hippocampus is not especially dedicated to allocentric memory and that lack of control for task difficulty may explain some of the deficits observed in previous studies. This account does not satisfactorily explain our findings, since task difficulty, as reflected in saccade accuracy, did not significantly differ between the three memory tasks in control subjects. Furthermore, analysis of saccade latencies in our patients provided no hint to an impairment of a decision process specific to the ALLO condition prior to execution of memory-guided eye movements.

It has been proposed that egocentric and allocentric representations are generated in parallel and that the behavioral context determines whether and how they interact [22]. Accordingly, a critical re-evaluation of studies investigating spatial memory in monkeys revealed that at least some of the discrepant results in the literature can consistently be explained by a failure to recognize and control for the fact that experimental subjects may flexibly use egocentric and allocentric representations in memory tasks [23]. Indeed, even in well-defined experimental settings such as virtual reality paradigms, the use of either representation may vary across subjects with concomitant recruitment of distinct neural substrates [33], [34]. Egocentric strategies may thus compensate for deficient allocentric memory in some tasks and in some subjects but not in others. In the experiments reported here, the availability of visual references was strictly controlled. In the EGO condition, the spatial cue was presented without any further visual references. Subjects were thus forced to encode the spatial cue relative to body coordinates, e.g. the distance and direction of the retinal cue image from the fovea. In the ALLO-EGO condition, subjects were provided the additional possibility to encode the spatial cue relative to a single spatial reference, i.e. the distance and direction of the spatial cue from the reference bar. In the ALLO condition, subjects' performance was entirely dependent on a correct representation of the geometric relationship between cue and reference bar.

At first glance, the impairment observed in our experiments seems to be at odds with normal findings in a previous study employing a similar task [35]. In this study, amnesic subjects with bilateral damage to the hippocampal formation were requested to remember the position of a dot on a horizontal sample bar. Similar to our ALLO task, a probe bar was presented at another location after a memory delay. Subjects were requested to locate the dot position on the probe bar. Normal performance of amnesic subjects with filled and unfilled delays of up to 12 seconds was taken as evidence against a role of the hippocampal formation in allocentric short-term memory [35]. However, this task differs importantly from our ALLO task, since subjects were free to shift their gaze to the sample and probe bars and thus to guide their responses by an egocentric representation, i.e. the retinal coordinates of the dot location. We are thus confident that the pattern of results obtained with our paradigms reflects the fact that certain aspects of spatial memory are disproportionally dependent on hippocampal integrity. The results in our ALLO-EGO condition further illustrate the fact that the brain may use egocentric strategies when allocentric representations are compromised, as soon as the behavioral context allows for it. This may explain some of the seeming contradictions between previous lesion studies on human spatial memory. For example, gradual viewpoint shifts in allocentric memory tasks may allow for compensatory egocentric strategies that are not efficient in instantaneous viewpoint shifts with otherwise similar stimulus material.

### Memory of geometric information in patients with hippocampal damage

Most tasks that have been devised to assess allocentric memory in humans make use of abstract or real-world-like spatial layouts with objects or landmarks that show a distinct spatial relationship with each other and the surface geometry of the layout. During the memory delay, the spatial relationship between subject and layout is manipulated. Then, memory of the spatial aspects of the layout is tested. Impaired performance in these tasks may thus either result from deficient representation of landmarks, their association to the layout or from the representation of geometric relationships *per se*. Previous behavioral research in patients with hippocampal lesions has not fully discriminated between the respective contributions of either function to performance. However, neurodevelopmental studies in various species including humans indicate that these aspects of spatial memory may be represented in distinct neural systems (see [24]–[26] for reviews). For example, in experimental environments, children initially reorient themselves mainly by using geometric information, while the ability to use landmark information develops later in life [36], [37]. A dissociation of these processes can also be found in reorienting healthy adults performing a concurrent verbal distraction task and in adult patients with neurodevelopmental disorders such as Williams syndrome [38], [39]. The common feature of our ALLO task and the tasks that were used in these studies, is that it assesses mapping of a spatial-geometric representation onto environmental information, and that this process cannot be fully achieved by egocentric strategies or by memory of non-geometric information only (i.e. landmarks). The selective deficit observed in the ALLO condition therefore suggests that memory even of simple geometric relationships is not sufficiently supported by extrahippocampal regions and is critically dependent on hippocampal integrity. This view is in line with findings from functional imaging studies showing that the right hippocampus stores locations relative to boundaries, but not to landmarks [40]. Furthermore, the corresponding hippocampal activations have been shown to increase parametrically with the amount of geometrical information required to represent a visuo-spatial scene, but not with scene complexity or task difficulty [41].

Few previous studies have investigated a possible lateralization of behavioral effects of unilateral damage to medial temporal lobe structures. Patients with extensive medial temporal lobe removals that include extrahippocampal structures such as the parahippocampal cortex have shown predominantly contralateral memory deficits both for visual and spatial material [42]–[44]. The methodology of most studies that employed viewpoint changes during the memory delay on patients with more restricted unilateral hippocampal damage did not allow for analysis of lateralization of deficits [e.g. 15], [33]. The bilaterality of the deficits observed in our patients may reflect the fact that the representation of view-point independent geometric information in the affected mediotemporal structures of our patients is distinct from more lateralized egocentric representation of space. Alternatively, it may result from a hemispheric asymmetry with a predominance of the right hemisphere for processing of visuospatial material. This hypothesis is however still controversial [18], [45]. A definite investigation of a spatial bias in our patients' deficit would require a different task design with a systematic mapping of the visual field with smaller stimulus displacements.

### Conclusion

Taken together, our findings are consistent with the hypothesis that the hippocampus is necessary to represent absolute spatial relationships, irrespective of the landmarks that are associated to them. Impairment of this function may contribute to deficient performance in more complex allocentric memory tasks. The simplicity of our stimulus material further shows that hippocampal involvement in spatial memory tasks is not solely driven by factors like memory load or the associative content of the stimulus material. However, similar to the geometric representation required in our ALLO condition, these factors may render a purely egocentric strategy based on transient and action-oriented representations insufficient. We therefore propose that a disproportionate hippocampal contribution to allocentric memory also reflects limits of egocentric representations that are generated in parallel and are presumably less dependent on the hippocampus.
